# Major Occupations and Private Insurance of Working Postpartum Women in Poverty in the United States, 2019

**DOI:** 10.1089/whr.2023.0042

**Published:** 2023-11-14

**Authors:** Bojung Seo, Hongmei Nan

**Affiliations:** ^1^ Department of Epidemiology, Richard M. Fairbanks School of Public Health, Indiana University, Indianapolis, Indiana, USA.; ^2^ Department of Global Health, Richard M. Fairbanks School of Public Health, Indiana University, Indianapolis, Indiana, USA.

**Keywords:** employee, health benefit plans, occupations, postpartum period, poverty, women, working

## Abstract

**Background:**

Although working postpartum women in poverty still have unmet medical needs, relevant research is lacking. Thus, we aimed to determine the five most frequent occupations of U.S. postpartum women in poverty and further examine whether the most frequent occupations are associated with poverty/being uninsured by an employer.

**Methods:**

This is a cross-sectional study. We included women who had a job and gave birth within the last 12 months from a 2019 American Community Survey Public Use Microdata Sample. To examine the associations between the most frequent occupations and being in poverty/uninsured through an employer/union, we used age- and race-adjusted and multivariable-adjusted logistic regression models.

**Results:**

A total of 14.3% of working postpartum women lived in poverty, and their most frequent major occupations were sales and related work, followed by food preparation and serving-related work, office and administrative support work, health care support work, and cleaning and ground maintenance. A total of 51.2% of women in the most frequent major occupations were uninsured through an employer/union. Compared with women in other occupations, women in the most frequent major occupations had fewer working hours and weeks that included paid leave. In particular, cleaners and ground maintenance workers and food preparation and serving-related workers were most likely to be in poverty and uninsured through an employer/union.

**Conclusions:**

Compared with other occupations, the most frequent occupations were more likely to be insecure and less likely to provide health insurance. Our U.S.-based study suggested that current policies regarding employee benefits needed to be improved especially for the most frequent major occupations.

## Introduction

The postpartum period, which covers 1 year after giving birth, is an important time in a woman's life that entails significant physiological and psychosocial changes.^1^ Mothers-to-be and new mothers need to recover from childbirth, adjust to changing hormones,^2^ and receive necessary maternal care services (*e.g.*, screening for gestational diabetes). Furthermore, newborn babies have an intense need to be with their mothers,^3^ since mother–infant interaction during this period substantially affects infants' language and cognitive development and emotional regulation.^4–6^

However, if postpartum women experience a household financial crisis, it may force them to work outside the home without professional aspirations. Sometimes this may require performing physically difficult work^7^ or foregoing meals and prenatal care.^8^ Working women who still face the financial strain of not having employment security, defined as having long-term employment contracts and employment relationships that avoid casualization,^9^ may suffer psychological distress,^10^ which also may negatively affect birth outcomes and the future health and behavior of the women's children.^11^ Hence, employer provision of health insurance and paid maternity leave to postpartum women will improve the long-term health of both mothers and infants. To be specific, having private insurance at delivery was associated with higher rates of well-woman visits, lower rates of emergency department visits, and lower odds of late prenatal care than Medicaid at delivery.^12^

In addition, perinatal women with private insurance where employment-based coverage consists of the largest share were significantly related to less unmet health care needs than those with public insurance.^13^ However, the Affordable Care Act (ACA) requires only large employers to offer health coverage to full-time workers and their dependents; this does not apply to small employers.^14^ Besides, mothers entitled to paid maternity leave beyond a few weeks' duration are more likely to have better mental and physical health, and their offspring, in turn, have a slightly reduced likelihood of infant death and an increased chance of secure maternal attachment, breastfeeding, and keeping up to date with vaccinations.^15,16^ Despite the clear necessity, only one relevant permanent policy, the Federal Employee Paid Leave Act, which became effective in October 2020, exists—and only for federal workers.^17^

Therefore, we aimed to investigate a research question: Are the most frequent occupations among postpartum women living in poverty associated with poverty or being uninsured by an employer? We hypothesized that compared with other occupations, the most frequent occupations among women in poverty would be more likely to be associated with poverty or being uninsured through an employer.

## Methods

### Study data and population

This cross-sectional study was a secondary analysis study using U.S. population-based national data. Specifically, we used the 2019 Public Use Microdata Sample (PUMS) data, a sample of full American Community Survey (ACS) microdata, which contains demographic, socioeconomic, and occupational information, including occupations, income-to-poverty ratio, and health insurance of respondents corresponding to ∼1% of the U.S. population.^18^ Response rates to the 2019 ACS were very high at 86.0% for housing unit and 90.9% for group quarters/persons.^19^ Furthermore, ACS PUMS data are of very high quality, given the measures to control biases or errors.^20^ In addition, to control measurement and processing error, ACS carefully monitored the work of interviewers, prepared field staff for their tasks, reinterviewed a sample of the households interviewed by computer-assisted personal interviewers, and imputed any remaining incomplete or inconsistent information in the collected data during the final content-editing phase.^20^

To confirm that our initial study data set was correctly downloaded and set up before conducting specific analyses, we compared our weighted frequencies and corresponding standard errors with PUMS estimates for user verification officially provided by the U.S. Census Bureau together with a comparison of record counts (https://www.census.gov/programs-surveys/acs/microdata/documentation.2019.html). The U.S. data include Washington, DC and all states, but excludes Puerto Rico. The study data were collected every day of the year 2019^21^ and analyzed between December 2021 and May 2022. We restricted our initial data set to women who had given birth and had jobs in the last 12 months.

Because most ACS PUMS responses were modified to protect the confidentiality of the survey respondents, the publicly available deidentified data used in this study were considered an exemption by the Institutional Review Board of Indiana University, and informed consent was not required. This study followed the Strengthening the Reporting of Observational Studies in Epidemiology reporting guidelines.

### Occupations

Occupation information was entered according to the 2018 standard occupational classification codes, which consists of 570 specific occupational categories and 25 major occupational groups. We defined the five-most frequent occupations among the 25 major occupational groups in the poverty group as “most frequent major occupations.” We also defined specific occupational categories with differences greater than 1.5% or less than −1.5% of weighted percentages between poverty and non-poverty groups as “specific jobs.”

### Poverty status

Poverty status was defined by an income-to-poverty ratio, also known as the Federal Poverty Level, determined by using income cutoffs that vary by family size and composition and additionally vary by age in the case of women living alone or with nonrelatives. The appropriate poverty thresholds were determined by multiplying the base-year poverty thresholds (1982) by the average of the monthly inflation factors for the 12 months preceding data collection. Accordingly, if a postpartum woman's total family income was below the threshold, the woman was considered to be in poverty; similarly, if an unrelated woman's total income was below the threshold, the woman was considered to be in poverty (*i.e.*, poverty = income-to-poverty ratio <100% vs. non-poverty = income-to-poverty ratio ≥100%). All poverty status-related variables cover information for the 12 months before data collection.

### Health insurance coverage

We also analyzed data of self-reported health insurance, including insurance through an employer/union, and private health insurance. Respondents with private health insurance had one of the following types of health insurance coverage: a plan provided through an employer/union, a plan purchased by an individual from a private company, or TRICARE or other military health care. Survey questions and more detailed explanations on the key variables of interest are described in Supplementary Data.

### Weeks worked including paid leave during the last 12 months

“Weeks worked” referred to weeks including paid vacation, paid sick leave, and military service. Therefore, if weeks worked during the past 12 months is longer in one occupation group than another, we considered that group to have more weeks worked during the past year and had better benefits related to paid leave.

### Statistical analysis

To accommodate the complex sample design of the ACS PUMS and calculate estimates representing the actual population, we used weighting variables of the PUMS weight and 80 replicate weights in all analyses. Continuous variables are presented as weighted mean (standard error) and categorical variables are presented as weighted frequency (weighted percentage) and its standard error. More details are provided in Supplementary Data.

To assess the hypothesis that the most frequent major occupations would be associated with poverty and being less insured by an employer/having any private health insurance, we performed age- and race-adjusted and multivariable-adjusted logistic regression, yielding adjusted odds ratios (aORs) and corresponding 95% confidence intervals (95% CIs). In the multivariable-adjusted logistic regression models, we further adjusted for age, race, citizenship status, marital status, ambulatory difficulty, and cognitive difficulty.

In addition, we calculated differences in weighted percentages between poverty and non-poverty groups regarding specific occupational categories of the most frequent major occupations (*i.e.*, weighted percentage of poverty group – weighted percentage of non-poverty group), from which we identified specific jobs with gaps in groups by poverty status. For the selected jobs, we further provided corresponding demographic and socioeconomic features according to poverty status to evaluate the hypothesis that specific jobs that were far more frequent in the poverty group would be less likely to be insured by an employer and have fewer working hours, including paid leave during the postpartum period.

All analyses were performed using SAS software (Unix 9.4; SAS Institute, Inc., Cary, NC, USA) and two-sided testing at a 5% level of statistical significance.

## Results

Among working postpartum women, 14.3% still lived in poverty despite working (Table 1). Compared with the non-poverty group, the women in the poverty group worked fewer hours (30.1 vs. 36.5) and weeks (33.8 vs. 44.8), which implied less paid leave. They were also less likely to be insured by an employer/union than those in the non-poverty group (17.5% vs. 71.7%). The private insurance gap was almost the same as the gap in insurance by an employer/union experienced by women in poverty (21.8% vs. 78.7%). The most frequent major occupations in the poverty group were sales and related work (16.6%), food preparation and serving-related work (16.0%), office and administrative support work (13.1%), health care support work (11.9%), and cleaning and ground maintenance work (7.1%).

**Table 1.** Demographic and Socio-Occupational Characteristics According to Poverty Status Among US Working Postpartum Women

Characteristics	Poverty		Non-poverty	
	***N*** (%)	SE	***N*** (%)	SE
Total women	387,864 (14.3)	8,655	2,333,940 (85.7)	19,940
Age (years)	28.1 (0.2)		31.3 (0.04)	
Race				
White	210,854 (54.4)	7,106	1,652,179 (70.8)	16,702
Black or African American	116,901 (30.1)	5,134	300,882 (12.9)	8,203
Others	60,109 (15.5)	3,651	380,879 (16.3)	8,889
Region				
Northeast	61,523 (15.9)	3,585	394,300 (16.9)	8,130
Midwest	88,073 (22.7)	3,804	526,516 (22.6)	10,056
South	165,382 (42.6)	5,682	865,053 (37.1)	11,098
West	72,886 (18.8)	3,406	548,071 (23.5)	8,617
Citizenship status				
Born in the US	327,518 (84.4)	7,994	1,915,010 (82.1)	17,963
Born in Puerto Rico, Guam, the US Virgin Islands, or the Northern Marianas	4,133 (1.1)	864	14,426 (0.6)	1,690
Born abroad of American parent(s)	2,163 (0.6)	545	30,325 (1.3)	2,768
US citizen by naturalization	14,851 (3.8)	1,484	168,612 (7.2)	5,664
Not a citizen of the US	39,199 (10.1)	3,018	205,567 (8.8)	6,694
Marital status				
Now married, spouse present	59,478 (15.3)	3,013	1,621,308 (69.5)	17,789
Now married, spouse absent	15,747 (4.1)	1,454	80,939 (3.5)	4,034
Widowed	2,013 (0.5)	639	5,466 (0.2)	871
Divorced	34,015 (8.8)	2,550	82,469 (3.5)	4,027
Separated	22,170 (5.7)	2,146	34,672 (1.5)	2,792
Never married	254,441 (65.6)	7,663	509,086 (21.8)	9,689
Ambulatory difficulty				
Yes	4,411 (1.1)	804	12,834 (0.5)	1,310
No	383,453 (98.9)	8,619	2,321,106 (99.5)	20,007
Cognitive difficulty				
Yes	14,087 (3.6)	1,714	28,976 (1.2)	2,201
No	373,777 (96.4)	8,431	2,304,964 (98.8)	20,256
Occupations^a^				
Sales and related workers	64,309 (16.6)	2,986	212,875 (9.1)	5,953
Food preparation and serving-related workers	61,959 (16.0)	3,641	120,859 (5.2)	4,093
Office and administrative support workers	50,891 (13.1)	3,156	328,051 (14.1)	6,616
Health care support workers	46,327 (11.9)	2,965	144,809 (6.2)	4,749
Cleaners and ground maintenance workers	27,539 (7.1)	2,360	42,038 (1.8)	2,689
Personal care and service workers	23,725 (6.1)	2,300	92,038 (3.9)	4,017
Transportation and material moving workers	23,592 (6.1)	2,012	60,435 (2.6)	3,921
Educational workers	22,304 (5.8)	2,294	261,690 (11.2)	6,803
Production workers	19,063 (4.9)	2,034	67,441 (2.9)	3,070
Medical workers	9,814 (2.5)	1,272	325,651 (14.0)	7,577
Managers	9,526 (2.5)	1,301	224,538 (9.6)	6,668
Farming, fishing, and forestry workers	4,578 (1.2)	1,084	7,134 (0.3)	1,514
Community and social service workers	4,563 (1.2)	900	68,245 (2.9)	3,618
Entertainment-field workers	4,105 (1.1)	901	56,333 (2.4)	2,725
Business workers	3,578 (0.9)	817	91,410 (3.9)	3,539
Protective service workers	3,458 (0.9)	856	25,122 (1.1)	2,351
Construction and related workers	2,388 (0.6)	634	8,286 (0.4)	1,360
Financial workers	1,473 (0.4)	566	58,355 (2.5)	3,122
Computer-field workers	1,299 (0.3)	607	42,725 (1.8)	3,162
Installation, maintenance, and repair workers	933 (0.2)	337	6,803 (0.3)	1,053
Legal workers	771 (0.2)	308	33,252 (1.4)	1,766
Scientists	616 (0.2)	314	31,566 (1.4)	2,047
Military workers	504 (0.1)	312	4,869 (0.2)	799
Extraction workers	295 (0.08)	286	119 (0.005)	122
Engineers	254 (0.07)	162	19,296 (0.8)	1,532
Usual hours worked per week past 12 months	30.1 (0.2)		36.5 (0.1)	
Weeks worked during past 12 months	33.8 (0.4)		44.8 (0.1)	
Income^b^				
Total person's income (dollars)	9,981.5 (215.8)		46,611.0 (384.4)	
Income-to-poverty ratio (%)	54.0 (0.7)		349.6 (1.1)	
Insurance through an employer/union				
Yes	67,927 (17.5)	3,717	1,673,589 (71.7)	18,541
No	319,937 (82.5)	7,854	660,351 (28.3)	10,127
Private health insurance coverage				
Yes	84,723 (21.8)	3,902	1,837,056 (78.7)	18,582
No	303,141 (78.2)	7,365	496,884 (21.3)	9,375

*Note:* Poverty (<100%) versus non-poverty (≥100%) was defined using income-to-poverty ratio. Continuous variables were presented with weighted mean (standard error of mean) and categorical variables with weighted frequency (weighted percentage). *p* < 0.0001.

^a^ Occupation information was collected by 2018 SOC codes.

^b^ Income variables were converted to 2019 constant dollars using inflation adjustment factor.

N, number; SE, standard error; SOC, standard occupational classification.

In Table 2, the most frequent major occupation group showed higher odds of being in poverty than other occupation groups (aOR: 1.86, 95% CI: 1.64–2.11). Except for office and administrative support workers, each of the most frequent major occupations was also significantly more likely to be associated with being in poverty compared with other occupations. In particular, cleaners and ground maintenance workers were more likely to live in poverty than workers in other occupations (aOR: 2.98, 95% CI: 2.44–3.62), followed by food preparation and serving-related workers (aOR: 2.51, 95% CI: 2.11–3.00).

**Table 2.** Adjusted Odds Ratios (95% Confidence Intervals) on Associations Between Most Frequent Major Occupations and Poverty Status

	Non-poverty	Poverty
Most frequent major occupations vs. others^a^	848,632/1,485,308	251,025/136,839
Age- and race-adjusted model^*^	1	2.59 (2.31–2.91)
Multivariable adjusted model^*^^,b^	1	1.86 (1.64–2.11)
Sales and related workers vs. others^a^	212,875/1,485,308	64,309/136,839
Age- and race-adjusted model^*^	1	2.39 (2.04–2.81)
Multivariable adjusted model^*^^,b^	1	1.74 (1.47–2.05)
Food preparation and serving-related workers vs. others^a^	120,859/1,485,308	61,959/136,839
Age- and race-adjusted model^*^	1	4.27 (3.58–5.10)
Multivariable adjusted model^*^^,b^	1	2.51 (2.11–3.00)
Office and administrative support workers vs. others^a^	328,051/1,485,308	50,891/136,839
Age- and race-adjusted model^**^	1	1.39 (1.17–1.66)
Multivariable adjusted model^b^	1	1.11 (0.91–1.35)
Health care support workers vs. others^a^	144,809/1,485,308	46,327/136,839
Age- and race-adjusted model^*^	1	2.52 (2.11–3.01)
Multivariable adjusted model^*^^,b^	1	2.01 (1.65–2.43)
Cleaners and ground maintenance workers vs. others^a^	42,038/1,485,308	27,539/136,839
Age- and race-adjusted model^*^	1	4.39 (3.64–5.28)
Multivariable adjusted model^*^^,b^	1	2.98 (2.44–3.62)

*Note:* Occupation information was collected using 2018 SOC codes. Most frequent major occupations are sales and related workers, food preparation and serving-related workers, office and administrative support workers, health care support workers, and cleaners and ground maintenance workers. Poverty (<100%) versus non-poverty (≥100%) was defined using income-to-poverty ratio.

^*^ *p* < 0.0001, ^**^p < 0.005.

^a^ Weighted frequency.

^b^ In multivariable model, we adjusted for age (continuous), race (white alone, black or African American alone, others), citizenship status (born in the US, born in Puerto Rico, Guam, the US virgin Islands, or the Northern Marianas, born abroad of American parents, US citizen by naturalization, not a citizen of the US), marital status (now married spouse present, now married spouse absent, widowed, divorced, separated, never married), ambulatory difficulty (yes, no), and cognitive difficulty (yes, no).

As shown in Table 3, 51.2% and 44.4% of women in the most frequent major occupations were uninsured through an employer/union or did not have private health insurance, respectively. Women in the most frequent major occupation group were significantly less likely to be insured *via* an employer/union (aOR: 2.09, 95% CI: 1.94–2.25) or to have no private insurance (aOR: 2.22, 95% CI: 2.03–2.42) than those in other occupation groups. In terms of each of the most frequent major occupations, cleaners and ground maintenance workers were more likely to be insured compared with other occupations (aOR: 3.64, 95% CI: 3.13–4.23; aOR: 4.07, 95% CI: 3.49–4.74), followed by food preparation and serving-related workers (aOR: 3.50, 95% CI: 3.05–4.03; aOR: 3.39, 95% CI: 2.96–3.87).

**Table 3.** Adjusted Odds Ratios (95% Confidence Intervals) on Associations Between Most Frequent Major Occupations and Insurance Through an Employer or Union/Private Health Insurance

	Insurance through an employer/union		Private health insurance	
	Yes	No	Yes	No
Most frequent major occupations vs. others^a,b^	536,303/1,205,213	563,353/416,934	611,013/1,310,766	488,644/311,381
Age- and race-adjusted model^*^	1	2.57 (2.40–2.75)	1	2.78 (2.57–3.01)
Multivariable adjusted model^*^^,c^	1	2.09 (1.94–2.25)	1	2.22 (2.03–2.42)
Sales and related workers vs. others^b^	132,884/1,205,213	144,300/416,934	151,418/1,310,766	125,766/311,381
Age- and race-adjusted model^*^	1	2.52 (2.25–2.82)	1	2.67 (2.37–3.01)
Multivariable adjusted model^*^^,c^	1	2.07 (1.83–2.34)	1	2.14 (1.86–2.47)
Food preparation and serving-related workers vs. others^b^	54,361/1,205,213	128,457/416,934	67,648/1,310,766	115,170/311,381
Age- and race-adjusted model^*^	1	5.60 (4.78–6.57)	1	5.72 (4.84–6.76)
Multivariable adjusted model^*^^,c^	1	3.86 (3.26–4.57)	1	3.74 (3.15–4.45)
Office and administrative support workers vs. others^b^	241,263/1,205,213	137,679/416,934	269,100/1,310,766	109,842/311,381
Age- and race-adjusted model^*^	1	1.42 (1.28–1.57)	1	1.43 (1.26–1.61)
Multivariable adjusted model^*^^,c^	1	1.25 (1.12–1.39)	1	1.23 (1.07–1.40)
Health care support workers vs. others^b^	91,069/1,205,213	100,067/416,934	102,842/1,310,766	88,294/311,381
Age- and race-adjusted model^*^	1	2.48 (2.18–2.81)	1	2.72 (2.38–3.10)
Multivariable adjusted model^*^^,c^	1	2.13 (1.86–2.43)	1	2.33 (2.01–2.70)
Cleaners and ground maintenance workers vs. others^b^	62,893/1,205,213	128,622/416,934	73,097/1,310,766	118,418/311,381
Age- and race-adjusted model^*^	1	4.92 (4.16–5.83)	1	5.60 (4.73–6.63)
Multivariable adjusted model^*^^,c^	1	3.64 (3.08–4.31)	1	4.08 (3.44–4.85)

*Note:* Occupation information was collected using 2018 SOC codes. Most frequent major occupations are sales and related workers, food preparation and serving-related workers, office and administrative support workers, health care support workers, and cleaners and ground maintenance workers.

^*^ *p* < 0.0001.

^a^ Among women with most frequent major occupations, 51.2% (563,353/1,099,656) and 44.4% (488,644/1,099,657) were uninsured through an employer/union and did not have private health insurance.

^b^ Weighted frequency.

^c^ In multivariable model, we adjusted for age (continuous), race (white alone, black or African American alone, others), citizenship status (born in the US, born in Puerto Rico, Guam, the US virgin Islands, or the Northern Marianas, born abroad of American parents, US citizen by naturalization, not a citizen of the US), marital status (now married spouse present, now married spouse absent, widowed, divorced, separated, never married), ambulatory difficulty (yes, no), and cognitive difficulty (yes, no).

Supplementary Table S1 shows that among specific occupational categories, cashiers, maids and housekeeping cleaners, janitors and building cleaners, waiters and waitresses, food preparation workers, fast food and counter workers, cooks, personal care aides, and nursing assistants were specific jobs far more frequent in the poverty group. In contrast, registered nurses and elementary and middle-school teachers were far more likely to be in the non-poverty group than the poverty group. Furthermore, some managers and medical workers such as chief executives and legislators and physicians were found only among women without poverty. Supplementary Tables S2 to S5 demonstrate that among women in poverty, those with specific jobs far more frequent in the poverty group had fewer weekly working hours and working weeks during the past 12 months, less insurance through an employer/union, and less private health insurance than those with other specific jobs.

## Discussion

Our data suggest that postpartum women working in most frequent major occupations, such as cleaners and ground maintenance workers and food preparation and serving-related workers, were less likely to have insurance by their employer/union, compared with women working in other occupations. In addition, the most frequent major occupations showed worse job security from their fewer working weeks that count paid leave than other occupations, implying that those are temporary or part-time jobs with worse job security.

Our data also showed that postpartum women in poverty in the most frequent major occupations worked fewer weeks, including paid leaves, probably because of fewer opportunities for maternity or family leave in those jobs. A 2019 national study regarding nonfederal public and private employers' employee benefits also reported similar results^22^; larger employers with more than 1,000 employees were more likely than smaller employers to provide paid parental leave, and larger employers with many higher income employees offered more paid parental leave than those with fewer higher income employees.^23^ Importantly, unlike other developed countries, the United States has no national standards on paid family or medical leave.^23^

In fact, despite efforts to enact a national paid family leave law, no permanent federal law for general employees has been passed.^23^ For example, The Family and Medical Insurance Leave Act, which provides partially paid parental leave for up to 12 weeks, and The Build Back Better Act, H.R. 5376, including health policies to provide almost all employees with partially paid family and medical leave for 4 weeks, have been debated but not yet passed.^23^ Furthermore, only 11 states offer paid family medical leave programs funded through employee-paid payroll taxes.^24^ Thus, because working postpartum women's access to paid maternity leave currently varies by state and employer, we need rapid and active consensus about a national standard policy for paid leave, given the clear potential benefits of paid parental or family leave, such as financial security and better maternal and infant health.^15^

Our study findings also indicated that women who worked in specific jobs far more frequently in the poverty group than in the non-poverty group had fewer working weeks including paid leave. This implied that those specific jobs were probably temporary or part-time jobs with worse job security than other specific jobs (*e.g.*, chief executives and legislators, and physicians). A recent study classified workers with any of four main occupations as working class (*i.e.*, retail and food jobs, blue-collar jobs, cubicle jobs, and caring jobs), all in agreement with our findings regarding the specific jobs far more frequent in the poverty group.^25^ Working-class women were more likely to be paid hourly wages and to be working through temporary agencies or a franchise system, and less likely to work in an office setting.^25^

Thus, working-class women depend on a meager pay and are more vulnerable to wage theft, unstable working schedules, and occupational and safety hazards.^25^ Such low job security may make both women in the working class and their children unhealthy, because women suffering from poor nourishment and mental distress caused by financial difficulty are at an elevated risk of adverse birth outcomes^26^ and long-term poor health of the child.^11^ Financial and mental hardship among working mothers could be addressed by employers' making them full-time, year-round employees. Thus, stable employment may protect maternal and child health.

We also observed lower rates of private health insurance, especially insurance through an employer/union, among postpartum women in the poverty group working in the most frequent major occupations. Typically, large employers offer more comprehensive coverage than smaller ones.^27^ Furthermore, in the pandemic era, there has been more competition among companies to hire talented workers,^28^ which in turn forced many small firms to improve employee pay and benefits.^28^ Because of those changes, the small companies became unable to afford to offer health care insurance.^28^ Furthermore, our data showed that the women in poverty uninsured by their employer were less likely to be covered by other private health insurances, such as a plan purchased by an individual from a private company.

Despite Medicaid options for women in poverty, still 2 million adults in poverty were uninsured because of state decisions not to expand Medicaid, indicating their income was above Medicaid eligibility but below the lower limit for marketplace premium tax credits.^29^ Moreover, the majority of workers covered by health coverage from their employer reported higher satisfaction with their coverage than those uninsured through their employer.^30^ However, the ACA is directed only at large employers.^14^ Thus, currently, there is no standard policy regarding employment-based health coverage for all employees. Moreover, deductibles for employment-based health coverage have risen sharply in recent years.^14^ Therefore, supportive policies are required to provide consistent levels of health insurance through employers/unions across all firms and reduce the burden of deductibles.

Our study has several strengths. First, our data came from a nationwide survey based on a high-quality complex sampling design, and we further performed user verification processes to accurately analyze the complex data, ensuring the accuracy and quality of our findings. Second, this survey collected multiple covariates, which enabled us to conduct more reliable analyses by adjusting for potential confounding factors.

We also acknowledge limitations. First, the private health insurance information collected covered the respondents at the time of the survey. Thus, we could not examine whether and how long they were covered by insurance through an employer/union or private health coverage, and whether there was any “churning” of health insurance during the postpartum period. Therefore, those parameters warrant future longitudinal studies that could examine those questions with more precise, long-term information. Second, we used weeks worked during the past year as an indirect measure of paid leave, which might lead to biased results. However, because the variable “weeks worked during the past year” included all cases of paid leave, the comparison of the length of time between two occupation groups itself may be enough to reflect job benefit/security related to paid leave during the postpartum period.

## Implications for Practice and/or Policy

Our study suggested that working postpartum women in poverty have relatively unstable jobs than those without poverty and the majority of them are single moms. Therefore, there is a need for social security systems to guarantee that working moms, especially single-mother families, in poverty could work as full-time, year-round employees. Furthermore, we observed that working postpartum women's access to paid maternity leave currently varies by state and employer; therefore, we need rapid and active consensus about a national standard policy for paid leave. Last but not least, supportive policies are required to provide consistent levels of health insurance through employers/unions across all firms and reduce the burden of deductibles.

## Conclusions

In summary, compared with other occupations, the most frequent major occupations of U.S. postpartum women in poverty were more likely to be insecure and less likely to include employer-based health coverage and have paid leave. In addition, the majority of working postpartum women in poverty in the most frequent major occupations were unmarried. Our study suggested that appropriate federal labor policies focusing on the specific jobs far more frequent in the poverty group should be enacted and passed to support paid parental or family leave and insurance through an employer/union as well as ensure financial security during the postpartum period. Particularly, for working single-mother households, more practical and specific policies are needed, such as a constitutional guarantee of flexible working or working from home. Future longitudinal studies are warranted for more thorough analyses.

## Supplementary Material

Supplemental data

Supplemental data

Supplemental data

Supplemental data

## Data Availability

The data sets we used and analyzed during the current study are publicly available on the ACS PUMS website: https://www2.census.gov/programs-surveys/acs/data/pums/2019/1-Year/

## Supplementary Material

[Supplementary-material SD4]

## Abbreviations Used

ACA: Affordable Care Act
ACS: American Community Survey
aORs: adjusted odds ratios
CIs: confidence intervals
PUMS: Public Use Microdata Sample
SE: standard error
SOC: standard occupational classification
