# Supplementary material for: Major Occupations and Private Insurance of Working Postpartum Women in Poverty in the United States, 2019
Source: Womens Health Rep (New Rochelle). 2023 Nov 14;4(1):497–505. doi: 10.1089/whr.2023.0042 (PMC10615075; doi:10.1089/whr.2023.0042)
Supplement: Supplemental data [file Suppl_Data.docx]

**Supplementary Material**

**Major occupations and private insurance of working postpartum women in poverty in the United States, 2019**

**1. Survey Questions for Employment, Weeks and Hours worked, Insurances, and Incomes^1^:**

1. **Employment**

*42. Descript of Employment: The next series of questions is about the type of employment this person had last week. If this person had more than one job, describe the one at which the most hours were worked. If this person did not work last week, describe the most recent employment in the past five years.*

- 1. Which one of the following best describes this person’s employment last week or the most recent employment in the past 5 years? *Mark (X) ONE box.*

**PRIVATE SECTOR EMPLOYEE**

**For-profit** company or organization

**Non-profit** organization (including tax-exempt and charitable organizations)

**GOVERNMENT EMPLOYEE**

**Local government** (for example: city or county school district)

**State government** (including state colleges/universities)

**Active duty** U.S. Armed Forces or Commissioned Corps

**Federal government** civilian employee

**SELF-EMPLOYED OR OTHER**

**Owner of non-incorporated** business, professional practice, or farm

**Owner of incorporated** business, professional practice, or farm

Worked **without pay** in a **for-profit** family business or farm for 15 hours or more per week

- 1. What was the name of this person’s employer, business, agency, or branch of the Armed Forces?
  2. What kind of business or industry was this?

*Include the main activity, product, or service provided at the location where employed. (For example: elementary school, residential construction)*

- 1. Was this mainly – *Mark (X) ONE box.*

manufacturing?

wholesale trade?

retail trade?

other (agriculture, construction, service, government, etc.)?

e. What was this person’s main occupation? *(For example: 4th grade teacher, entry-level plumber)*

f. Describe this person’s most important activities or duties. *(For example: instruct and evaluate students and create lesson plans, assemble and install pipe sections and review building plans for work details)*

1. **Weeks worked during the last 12 months that count paid leave and working hours**

40-a. During the PAST 12 MONTHS (52 weeks), did this person work EVERY week? Count paid vacation, paid sick leave, and military service as work.

Yes -> *SKIP to question 41*

No

40-b. During the PAST 12 MONTHS (52 weeks), how many WEEKS did this person work? Include paid time off and include weeks when the person only worked for a few hours.

41. During the PAST 12 MONTHS, in the WEEKSWORKED, how many hours did this person usually work each WEEK?

Usual hours worked each WEEK ( )

1. **Health Insurance Coverage**

16. Is this person CURRENTLY covered by any of the following types of health insurance or health coverage plans? Mark "Yes" or "No" for EACH type of coverage in items a – h.

1. Insurance through a current or former employer or union (of this person or another family member): Yes or No
2. Insurance purchased directly from an insurance company (by this person or another family member): Yes or No
3. Medicare, for people 65 and older, or people with certain disabilities: Yes or No
4. Medicaid, Medical Assistance, or any kind of government-assistance plan for those with low incomes or a disability: Yes or No
5. TRICARE or other military health care: Yes or No
6. VA (enrolled for VA health care): Yes or No
7. Indian Health Service: Yes or No
8. Any other type of health insurance or health coverage plan: Yes or No

– Specify (open-ended)

1. **Incomes in the past 12 months**

43. INCOME IN THE PAST 12 MONTHS

Mark (X) the "Yes" box for each type of income this person received, and give your best estimate of the TOTAL AMOUNT during the PAST 12 MONTHS. (NOTE: The "past 12 months" is the period from today’s date one year ago up through today.) Mark (X) the "No" box to show types of income NOT received. If net income was a loss, mark the "Loss" box to the right of the dollar amount. For income received jointly, report the appropriate share for each person – or, if that’s not possible, report the whole amount for only one person and mark the "No" box for the other person.

1. Wages, salary, commissions, bonuses, or tips from all jobs. Report amount before deductions for taxes, bonds, dues, or other items: Yes or No

– Specify total amount for past 12 months ($ )

1. Self-employment income from own nonfarm businesses or farm businesses, including proprietorships and partnerships. Report NET income after business expenses: Yes or No

– Specify total amount for past 12 months ($ )

Was that loss?: Yes or No

1. Interest, dividends, net rental income, royalty income, or income from estates and trusts. Report even small amounts credited to an account: Yes or No

– Specify total amount for past 12 months ($ )

Was that loss?: Yes or No

1. Social Security or Railroad Retirement: Yes or No

– Specify total amount for past 12 months ($ )

1. Supplemental Security Income (SSI): Yes or No

– Specify total amount for past 12 months ($ )

1. Any public assistance or welfare payments from the state or local welfare office: Yes or No

– Specify total amount for past 12 months ($ )

1. Retirement income, pensions, survivor or disability income. Include income from a previous employer or union, or any regular withdrawals or distributions from IRA, Roth IRA, 401(k), 403(b), or other accounts specifically designed for retirement. Do not include Social Security: Yes or No

– Specify total amount for past 12 months ($ )

1. Any other sources of income received regularly such as Veterans’ (VA) payments, unemployment compensation, child support or alimony. Do NOT include lump sum payments such as money from an inheritance or the sale of a home: Yes or No

– Specify total amount for past 12 months ($ )

1. **Person’s total incomes in the past 12 months**

44. What was this person’s total income during the PAST 12 MONTHS? Add entries in questions 2a to 2h; subtract any losses. If net income was a loss, enter the amount and mark (X) the "Loss" box next to the dollar amount:

Is that None? Otherwise specify total amount for past 12 months ($ )

Was that loss?: Yes or No

**Note:** Overall, total personal income examined in this study was a sum added altogether with total amounts of wages, salary, commissions, bonuses, or tips from all jobs before deductions for taxes, bonds, dues, or other items, total self-employment net income from own nonfarm businesses or farm businesses after business expenses, total amounts of interest, dividends, net rental income, royalty income, or income from estates and trusts that were credited to an account, total amounts of social security or railroad retirement, total amounts of supplemental security income, total amounts of any public assistance or welfare payments from the state or local welfare office, total amounts of retirement income, pensions, survivor or disability income including incomes from a previous employer or union, or any regular withdrawals or distributions from IRA, Roth IRA, 401(k), 403(b), or other accounts specifically designed for retirement but not including social security, and total amounts of any other sources of income received regularly such as Veterans’ payments, unemployment compensation, child support or alimony, not including lump sum payments such as money from an inheritance or the sale of a home; any losses were subtracted from the total person’s incomes thereafter.

**2. Occupations measure:**

Occupational data were derived primarily from answers to questions (respondent’s main occupation and most important activities or duties) asked of all persons aged 15 and over who had worked in the past 5 years. For an employed individual, the person’s job during the previous week was collected. For a woman who worked two or more jobs, a job where the person worked the greatest number of hours was included. In terms of lifelong unemployed people and people who were not employed but reported having a job within the last five years at the time of the survey, their last jobs were included.

**3. Estimates of Poverty^2^:**

**How Poverty Is Calculated?**

Poverty status was defined by income-to-poverty ratio determined by using income cutoffs that vary by family size and composition and additionally vary by age in the case of females living alone or with nonrelatives. If a peripartum female’s total family income was less than the appropriate threshold, then the female was considered to be in poverty; similarly, if an unrelated female’s total income was less than the appropriate threshold, then the female was considered to be in poverty. The appropriate poverty thresholds were determined by multiplying the base-year poverty thresholds (1982) by the average of the monthly inflation factors for the 12 months preceding the data collection.

Following the Office of Management and Budget’s (OMB) Statistical Policy Directive 14, the U.S. Census Bureau uses a set of dollar value thresholds that vary by family size and composition to determine who is in poverty.

**Poverty Thresholds for 2019 by Size of Family and Number of Related Children Under 18 Years (in dollars):**

|  | | | | | | | | | |
| --- | --- | --- | --- | --- | --- | --- | --- | --- | --- |
| **Size of family unit** | **Related children under 18 years** | | | | | | | | |
|  | **None** | **One** | **Two** | **Three** | **Four** | **Five** | **Six** | **Seven** | **Eight or more** |
| One person (unrelated individual): |  |  |  |  |  |  |  |  |  |
| Under age 65 | 13,300 |  |  |  |  |  |  |  |  |
| Aged 65 and older | 12,261 |  |  |  |  |  |  |  |  |
| Two people: |  |  |  |  |  |  |  |  |  |
| Householder under age 65 | 17,120 | 17,622 |  |  |  |  |  |  |  |
| Householder aged 65 and older | 15,453 | 17,555 |  |  |  |  |  |  |  |
| Three people | 19,998 | 20,578 | 20,598 |  |  |  |  |  |  |
| Four people | 26,370 | 26,801 | 25,926 | 26,017 |  |  |  |  |  |
| Five people | 31,800 | 32,263 | 31,275 | 30,510 | 30,044 |  |  |  |  |
| Six people | 36,576 | 36,721 | 35,965 | 35,239 | 34,161 | 33,522 |  |  |  |
| Seven people | 42,085 | 42,348 | 41,442 | 40,811 | 39,635 | 38,262 | 36,757 |  |  |
| Eight people | 47,069 | 47,485 | 46,630 | 45,881 | 44,818 | 43,470 | 42,066 | 41,709 |  |
| Nine people or more | 56,621 | 56,895 | 56,139 | 55,503 | 54,460 | 53,025 | 51,727 | 51,406 | 49,426 |
| Source: U.S. Census Bureau. | | | | | | | | | |

If a family’s total money income is less than the applicable threshold, then that family and every individual in it are considered to be in poverty. The official poverty thresholds are updated annually for inflation using the Consumer Price Index for All Urban Consumers (CPI-U). The official poverty definition uses money income before taxes or tax credits and excludes capital gains and noncash benefits (such as Supplemental Nutrition Assistance Program benefits and housing assistance). The thresholds do not vary geographically. *Example:* Suppose Family A comprises five people: two children, their mother, their father, and their great-aunt. Family A’s poverty threshold in 2019 is $31,275. Each member of Family A had the following income in 2019: Mother $11,000 Father $11,000 Great-aunt $10,000 First child 0 Second child 0 Total: $32,000

Since their total family income ($32,000) was higher than their threshold ($31,275), Family A would not be considered “in poverty.”

While the thresholds, in some sense, represent the needs of families, they should be interpreted as a statistical yardstick rather than as a complete description of what people and families need to live. Many government assistance programs use different income eligibility cutoffs. While official poverty rates and the number of people or families in poverty are important, other poverty indicators are considered in the section “Depth of Poverty Measures,” and another approach to setting thresholds and defining resources is discussed in the section “Supplemental Poverty Measure.”

For a history of the official poverty measure, see “Poverty: The History of the Official Poverty Measure” available at <www.census.gov/topics/income-poverty/poverty/about/history-of-the-poverty-measure.html> or “The Development of the Orshansky Poverty Thresholds and Their Subsequent History as the Official U.S. Poverty Measure” by Gordon M. Fisher, available at <www.census.gov/library/working-papers/1997/demo/fisher-02.html>.

**Weighted Average Thresholds:**

Since some data users want a summary of the 48 thresholds to get a general sense of the “poverty line,” the following table provides the weighted average thresholds for 2019. The weighted average thresholds are based on the relative number of unrelated individuals and primary families of each size and composition and are not used in computing poverty estimates (a primary family is a group of two or more people, one of whom is the householder, related by birth, marriage, or adoption and residing together. All such people including related subfamily members are considered as members of one family).

**Weighted Average Poverty Thresholds in 2019:**

| **Size of family unit** | **Dollars** |
| --- | --- |
| One person | 13,011 |
| Two people | 16,521 |
| Three people | 20,335 |
| Four people | 26,172 |
| Five people | 31,021 |
| Six people | 35,129 |
| Seven people | 40,016 |
| Eight people | 44,461 |
| Nine people or more | 52,875 |

Source: U.S. Census Bureau.

**4. Detailed methodologies using weighting variables:**

The ACS PUMS is a complex sample design with its weighting variables to represent the actual population. Weights from PUMS person records were created from the final full ACS weight, the PUMS subsampling factors (*i.e.,* the sampling intervals used to sample the PUMS group quarters or housing units/household person records within a state), and ratio-estimate factors used to bring the PUMS estimates into closer agreement with the published ACS estimates. We used PUMS weighting variables in all analyses to calculate not only weighted estimates, but also accurate measures of uncertainty of the weighted estimates (*i.e.,* SEs). Specifically, we used person’s weight named PWGTP, for generation of the statistics on individuals, and 80 replicate weights named PWGTP1 to PWGTP80, for estimation of the SEs. To obtain the SE, we used a formula of successive difference replication SE: $\sqrt{VAR \left( x \right)}=\sqrt{\frac{4}{80}\sum_{r=1}^{80} {(x_{r}-x)}^{2}}$ ($x_{r}$is a r^th^ replicate estimate, and x is the full PUMS weighted estimate).

**References**

1. ACS Questionnaire 2019. Census.gov; <https://www2.census.gov/programs-surveys/acs/methodology/questionnaires/2019/quest19.pdf>

2. Jessica Semega MK, Emily A. Shrider, and John F. Creamer. *Income and Poverty in the United States: 2019* 2020. *Current Population Reports*. September 15, 2020. <https://www.census.gov/content/dam/Census/library/publications/2020/demo/p60-270.pdf>

3. Status of State Action on the Medicaid Expansion Decision. Kaiser Family Foundation. Updated July 21, 2022. Accessed August 2, 2022, 2022. <https://www.kff.org/health-reform/state-indicator/state-activity-around-expanding-medicaid-under-the-affordable-care-act/?currentTimeframe=0&sortModel=%7B%22colId%22:%22Location%22,%22sort%22:%22asc%22%7D>
