# Supplementary material for: Major Occupations and Private Insurance of Working Postpartum Women in Poverty in the United States, 2019
Source: Womens Health Rep (New Rochelle). 2023 Nov 14;4(1):497–505. doi: 10.1089/whr.2023.0042 (PMC10615075; doi:10.1089/whr.2023.0042)
Supplement: Supplemental data [file Suppl_TableS1.docx]

**Table S1. Specific jobs of most frequent major occupations ^a, b^ by poverty status ^c^ among US working postpartum women ^d, e^**

|  | **Poverty** | | **Non-poverty** | | **Difference of % ^f^** |
| --- | --- | --- | --- | --- | --- |
| **Characteristics** | **N (%)** | **SE** | **N (%)** | **SE** |  |
| **Sales and related workers** | 64,309 (22.0) | 2,986 | 212,875 (12.8) | 5,953 |  |
| SAL-First-Line Supervisors Of Retail Sales Workers | 6,675 (2.3) | 1,117 | 45,890 (2.8) | 3,121 | -0.48 |
| SAL-First-Line Supervisors Of Non-Retail Sales Workers | 430 | 206 | 10,809 (0.7) | 1,391 | -0.5 |
| SAL-Cashiers | 39,797 (13.6) | 2,293 | 62,658 (3.8) | 3,841 | 9.82 |
| SAL-Counter And Rental Clerks | 232 (0.08) | 185 | 1,206 (0.07) | 579 | 0.01 |
| SAL-Parts Salespersons | 170 (0.06) | 134 | 459 (0.03) | 287 | 0.03 |
| SAL-Retail Salespersons | 10,340 (3.5) | 1,238 | 36,798 (2.2) | 2,789 | 1.32 |
| SAL-Advertising Sales Agents | 54 (0.02) | 56 | 2,571 (0.2) | 690 | -0.14 |
| SAL-Insurance Sales Agents | 610 (0.2) | 311 | 11,511 (0.7) | 1,247 | -0.48 |
| SAL-Securities, Commodities, And Financial Services Sales Agents |  |  | 1,907 (0.1) | 483 | -0.11 |
| SAL-Travel Agents | 91 (0.03) | 91 | 1,194 (0.07) | 516 | -0.04 |
| SAL-Sales Representatives Of Services, Except Advertising, Insurance, Financial Services, And Travel | 747 (0.3) | 424 | 7,396 (0.4) | 1,101 | -0.19 |
| SAL-Sales Representatives, Wholesale And Manufacturing | 443 (0.2) | 204 | 10,646 (0.6) | 988 | -0.49 |
| SAL-Models, Demonstrators, And Product Promoters | 448 (0.2) | 307 | 170 (0.01) | 127 | 0.14 |
| SAL-Real Estate Brokers And Sales Agents | 1,114 (0.4) | 528 | 12,226 (0.7) | 1,423 | -0.36 |
| SAL-Telemarketers | 936 (0.3) | 418 | 1,399 (0.08) | 490 | 0.24 |
| SAL-Door-To-Door Sales Workers, News And Street Vendors, And Related Workers | 1,431 (0.5) | 488 | 1,981 (0.1) | 433 | 0.37 |
| SAL-Sales And Related Workers, All Other | 791 (0.3) | 503 | 4,054 (0.2) | 791 | 0.03 |
| **Food preparation and serving related workers** | 61,959 (21.2) | 3,641 | 120,859 (7.3) | 4,093 |  |
| EAT-Chefs And Head Cooks | 782 (0.3) | 400 | 4,047 (0.2) | 857 | 0.02 |
| EAT-First-Line Supervisors Of Food Preparation And Serving Workers | 2,472 (0.8) | 643 | 9,845 (0.6) | 1,217 | 0.25 |
| EAT-Cooks | 13,306 (4.5) | 1,490 | 19,695 (1.2) | 2,041 | 3.36 |
| EAT-Food Preparation Workers | 7,901 (2.7) | 1,249 | 13,843 (0.8) | 1,451 | 1.87 |
| EAT-Bartenders | 4,117 (1.4) | 927 | 8,847 (0.5) | 1,261 | 0.87 |
| EAT-Fast Food And Counter Workers | 6,223 (2.1) | 1,233 | 8,821 (0.5) | 1,415 | 1.6 |
| EAT-Waiters And Waitresses | 21,872 (7.5) | 2,249 | 43,573 (2.6) | 2,494 | 4.85 |
| EAT-Food Servers, Nonrestaurant | 1,635 (0.6) | 587 | 2,563 (0.2) | 599 | 0.4 |
| EAT-Dining Room And Cafeteria Attendants And Bartender Helpers | 1,078 (0.4) | 416 | 2,051 (0.1) | 595 | 0.24 |
| EAT-Dishwashers | 755 (0.3) | 534 | 2,276 (0.1) | 689 | 0.12 |
| EAT-Hosts And Hostesses, Restaurant, Lounge, And Coffee Shop | 1,740 (0.6) | 585 | 5,298 (0.3) | 1,116 | 0.28 |
| EAT-Food Preparation And Serving Related Workers, All Other | 78 (0.03) | 78 |  |  | 0.03 |
| **Office and administrative support workers** | 50,891 (17.4) | 3,156 | 328,051 (19.8) | 6,616 |  |
| OFF-First-Line Supervisors Of Office And Administrative Support Workers | 1,696 (0.6) | 562 | 19,560 (1.2) | 1,611 | -0.6 |
| OFF-Switchboard Operators, Including Answering Service | 76 (0.03) | 79 | 1,111 (0.07) | 664 | -0.04 |
| OFF-Telephone Operators | 471 (0.2) | 321 | 963 (0.06) | 415 | 0.1 |
| OFF-Communications Equipment Operators, All Other |  |  | 382 (0.02) | 231 | -0.02 |
| OFF-Bill And Account Collectors | 617 (0.2) | 377 | 2,898 (0.2) | 679 | 0.04 |
| OFF-Billing And Posting Clerks | 976 (0.3) | 439.0286 | 10,543 (0.6) | 1,391 | -0.3 |
| OFF-Bookkeeping, Accounting, And Auditing Clerks | 1,377 (0.5) | 395 | 17,453 (1.1) | 1,695 | -0.58 |
| OFF-Payroll And Timekeeping Clerks |  |  | 2,890 (0.2) | 522 | -0.17 |
| OFF-Procurement Clerks |  |  | 350 (0.02) | 171 | -0.02 |
| OFF-Tellers | 1,541 (0.5) | 509 | 11,527 (0.7) | 1,439 | -0.17 |
| OFF-Other Financial Clerks |  |  | 1,628 (0.1) | 372 | -0.1 |
| OFF-Court, Municipal, And License Clerks | 151 (0.05) | 95 | 721 (0.04) | 230 | 0.01 |
| OFF-Credit Authorizers, Checkers, And Clerks | 142 (0.05) | 149 | 1,500 (0.1) | 547 | -0.04 |
| OFF-Customer Service Representatives | 11,372 (3.9) | 1,599 | 58,045 (3.5) | 3,461 | 0.39 |
| OFF-Eligibility Interviewers, Government Programs | 724 (0.2) | 526 | 2,023 (0.1) | 552 | 0.13 |
| OFF-File Clerks | 566 (0.2) | 270 | 3,422 (0.2) | 908 | -0.01 |
| OFF-Hotel, Motel, And Resort Desk Clerks | 2,303 (0.8) | 634 | 3,469 (0.2) | 1,049 | 0.58 |
| OFF-Interviewers, Except Eligibility And Loan | 1,685 (0.6) | 575 | 2,616 (0.2) | 540 | 0.42 |
| OFF-Library Assistants, Clerical | 1,135 (0.4) | 526 | 373 (0.02) | 177 | 0.37 |
| OFF-Loan Interviewers And Clerks |  |  | 3,626 (0.2) | 911 | -0.22 |
| OFF-New Accounts Clerks |  |  | 108 (0.007) | 78 | -0.01 |
| OFF-Human Resources Assistants, Except Payroll And Timekeeping | 71 (0.02) | 77 | 2,305 (0.1) | 550 | -0.11 |
| OFF-Receptionists And Information Clerks | 8,110 (2.8) | 1,465 | 43,098 (2.6) | 2,646 | 0.18 |
| OFF-Reservation And Transportation Ticket Agents And Travel Clerks | 149 (0.05) | 146.967 | 1,383 (0.08) | 432 | -0.03 |
| OFF-Correspondence Clerks And Order Clerks |  |  | 1,594 (0.1) | 566 | -0.1 |
| OFF-Other Information And Records Clerks | 532 (0.2) | 400 | 1,958 (0.1) | 553 | 0.06 |
| OFF-Couriers And Messengers | 682 (0.2) | 385 | 1,680 (0.1) | 543 | 0.13 |
| OFF-Public Safety Telecommunicators | 467 (0.2) | 230 | 2,548 (0.2) | 649 | 0.01 |
| OFF-Dispatchers, Except Police, Fire, And Ambulance | 418 (0.1) | 370 | 2,239 (0.1)_ | 662 | 0.01 |
| OFF-Postal Service Clerks |  |  | 1,015 (0.06) | 490 | -0.06 |
| OFF-Postal Service Mail Carriers | 286 (0.1) | 224 | 3,932 (0.2) | 984 | -0.14 |
| OFF-Postal Service Mail Sorters, Processors, And Processing Machine Operators |  |  | 376 (0.02) | 246 | -0.02 |
| OFF-Production, Planning, And Expediting Clerks | 522 (0.2) | 365 | 4,562 (0.3) | 838 | -0.1 |
| OFF-Shipping, Receiving, And Inventory Clerks | 2,517 (0.9) | 907 | 6,575 (0.4) | 1,376 | 0.46 |
| OFF-Weighers, Measurers, Checkers, And Samplers, Recordkeeping | 94 (0.03) | 118 | 1,210 (0.07) | 405 | -0.04 |
| OFF-Executive Secretaries And Executive Administrative Assistants | 81 (0.03) | 56 | 4,780 (0.3) | 1,167 | -0.26 |
| OFF-Legal Secretaries And Administrative Assistants |  |  | 1,685 (0.1) | 709 | -0.1 |
| OFF-Medical Secretaries And Administrative Assistants |  |  | 1,516 (0.09) | 403 | -0.09 |
| OFF-Secretaries And Administrative Assistants, Except Legal, Medical, And Executive | 6714 | 1,285 | 45,346 (2.7) | 2,707 | -0.44 |
| OFF-Data Entry Keyers | 539 | 251.997 | 4,449 (0.3) | 883 | -0.08 |
| OFF-Word Processors And Typists | 89 | 92 | 942 (0.06) | 394 | -0.03 |
| OFF-Insurance Claims And Policy Processing Clerks | 43 | 48 | 5,007 (0.3) | 978 | -0.29 |
| OFF-Mail Clerks And Mail Machine Operators, Except Postal Service | 458 | 286.9563 | 312 (0.02) | 270 | 0.14 |
| OFF-Office Clerks, General | 2865 | 711 | 27,140 (1.6) | 2,267 | -0.66 |
| OFF-Office Machine Operators, Except Computer | 54 | 57 | 832 (0.05) | 375 | -0.03 |
| OFF-Proofreaders And Copy Markers |  |  | 90 (0.005) | 94 | -0.01 |
| OFF-Statistical Assistants |  |  | 296 (0.02) | 212 | -0.02 |
| OFF-Other Office And Administrative Support Workers | 1368 | 478 | 15,973 (1.0) | 1,735 | -0.49 |
| **Healthcare support workers** | 46,327 (15.8) | 2,965 | 144,809 (8.7) | 4,749 |  |
| HLS-Home Health Aides | 5,469 (1.9) | 1,088 | 10,581 (0.6) | 1,559 | 1.23 |
| HLS-Personal Care Aides | 14,706 (5.0) | 1,950 | 26,913 (1.6) | 2,334 | 3.4 |
| HLS-Nursing Assistants | 17,348 (5.9) | 1,942 | 45,012 (2.7) | 3,269 | 3.22 |
| HLS-Orderlies And Psychiatric Aides | 1,122 (0.4) | 718 | 787 (0.05) | 460 | 0.34 |
| HLS-Occupational Therapy Assistants And Aides | 50 (0.02) | 54 | 2,665 (0.2) | 655 | -0.14 |
| HLS-Physical Therapist Assistants And Aides | 35 (0.01) | 43 | 5,106 (0.3) | 988 | -0.3 |
| HLS-Massage Therapists | 815 (0.3) | 473 | 5,467 (0.3) | 865 | -0.05 |
| HLS-Dental Assistants | 1,478 (0.5) | 482 | 14,573 (0.9) | 1,826 | -0.37 |
| HLS-Medical Assistants | 2,880 (1.0) | 767 | 23,453 (1.4) | 2,024 | -0.43 |
| HLS-Medical Transcriptionists | 25 (0.01) | 27 | 487 (0.03) | 212 | -0.02 |
| HLS-Pharmacy Aides | 294 (0.1) | 262 | 987 (0.06) | 319 | 0.04 |
| HLS-Veterinary Assistants And Laboratory Animal Caretakers | 179 (0.06) | 141 | 1,227 (0.07) | 382 | -0.01 |
| HLS-Phlebotomists | 631 (0.2) | 296 | 4,720 (0.3) | 973 | -0.07 |
| HLS-Other Healthcare Support Workers | 1,295 (0.4) | 504 | 2,831 (0.2) | 687 | 0.27 |
| **Cleaners and grounds maintenance workers** | 27,539 (9.4) | 2,360 | 42,038 (2.5) | 2,689 |  |
| CLN-First-Line Supervisors Of Housekeeping And Janitorial Workers | 574 (0.2) | 361 | 1,010 (0.06) | 380 | 0.14 |
| CLN-First-Line Supervisors Of Landscaping, Lawn Service, And Groundskeeping Workers | 242 (0.08) | 207 | 624 (0.04) | 382 | 0.05 |
| CLN-Maids And Housekeeping Cleaners | 18,217 (6.2) | 1,830 | 22,813 (1.4) | 1,972 | 4.85 |
| CLN-Janitors And Building Cleaners | 7,166 (2.4) | 1,248 | 15,325 (0.9) | 1,872 | 1.53 |
| CLN-Landscaping And Groundskeeping Workers | 1,340 (0.5) | 654.9676 | 2,185 (0.1) | 876 | 0.33 |
| CLN-Tree Trimmers And Pruners |  |  | 81 (0.005) | 81 | 0 |
| **Managers** | 9,526 (3.3) | 1,301 | 224,538 (13.5) | 6,668 |  |
| MGR-General And Operations Managers | 52 (0.02) | 55 | 15,571 (0.9) | 1,673 | -0.92 |
| MGR-Chief Executives And Legislators |  |  | 7,775 (0.5) | 1,212 | -0.47 |
| MGR-Advertising And Promotions Managers |  |  | 851 (0.05) | 261 | -0.05 |
| MGR-Marketing Managers | 239 (0.08) | 178 | 17,592 (1.1) | 1,379 | -0.98 |
| MGR-Sales Managers |  |  | 6,262 (0.4) | 1,107 | -0.38 |
| MGR-Public Relations And Fundraising Managers |  |  | 3,545 (0.2) | 851 | -0.21 |
| MGR-Administrative Services Managers |  |  | 1,113 (0.07) | 338 | -0.07 |
| MGR-Facilities Managers |  |  | 245 (0.01) | 187 | -0.01 |
| MGR-Computer And Information Systems Managers | 74 (0.03) | 74 | 6,900 (0.4) | 1,353 | -0.39 |
| MGR-Financial Managers | 124 (0.04) | 97 | 27,104 (1.6) | 1,942 | -1.59 |
| MGR-Industrial Production Managers |  |  | 1,593 (0.1) | 476 | -0.1 |
| MGR-Purchasing Managers |  |  | 1,996 (0.1) | 518 | -0.12 |
| MGR-Transportation, Storage, And Distribution Managers | 69 (0.02) | 74 | 793 (0.05) | 310 | -0.02 |
| MGR-Compensation And Benefits Managers |  |  | 71 (0.004) | 51 | 0 |
| MGR-Human Resources Managers | 170 (0.06) | 171 | 6,887 (0.4) | 998 | -0.36 |
| MGR-Training And Development Managers |  |  | 977 (0.06) | 304 | -0.06 |
| MGR-Farmers, Ranchers, And Other Agricultural Managers | 150 (0.05) | 106 | 3,199 (0.2) | 820 | -0.14 |
| MGR-Construction Managers | 87 (0.03) | 86 | 1,915 (0.1) | 548 | -0.09 |
| MGR-Education And Childcare Administrators | 229 (0.08) | 166 | 17,856 (1.1) | 1,566 | -1 |
| MGR-Architectural And Engineering Managers |  |  | 763 (0.05) | 288 | -0.05 |
| MGR-Food Service Managers | 3,816 (1.3) | 791 | 18,755 (1.1) | 2,147 | 0.17 |
| MGR-Entertainment And Recreation Managers |  |  | 475 (0.03) | 215 | -0.03 |
| MGR-Lodging Managers | 352 (0.1) | 268 | 2,974 (0.2) | 817 | -0.06 |
| MGR-Medical And Health Services Managers | 192 (0.07) | 160 | 15,215 (0.9) | 1,347 | -0.85 |
| MGR-Natural Sciences Managers |  |  | 218 (0.01) | 133 | -0.01 |
| MGR-Property, Real Estate, And Community Association Managers | 330 (0.1) | 209 | 8,534 (0.5) | 976 | -0.4 |
| MGR-Social And Community Service Managers | 425 (0.1) | 296 | 8,111 (0.5) | 1,135 | -0.34 |
| MGR-Emergency Management Directors |  |  | 76 (0.005) | 81 | 0 |
| MGR-Other Managers | 3,217 (1.1) | 829 | 47,172 (2.8) | 2,851 | -1.74 |
| **Educational workers** | 22,304 (7.6) | 2,294 | 261,690 (15.8) | 6,803 |  |
| EDU-Postsecondary Teachers | 840 (0.3) | 477 | 23,500 (1.4) | 2,002 | -1.13 |
| EDU-Preschool And Kindergarten Teachers | 5,231 (1.8) | 984 | 39,088 (2.4) | 2,997 | -0.57 |
| EDU-Elementary And Middle School Teachers | 3,460 (1.2) | 787 | 96,854 (5.8) | 3,784 | -4.65 |
| EDU-Secondary School Teachers | 1,245 (0.4) | 520 | 33,269 (2.0) | 2,038 | -1.58 |
| EDU-Special Education Teachers | 63 (0.02) | 63 | 14,083 (0.8) | 1,359 | -0.83 |
| EDU-Tutors | 256 (0.09) | 175 | 2,558 (0.2) | 604 | -0.07 |
| EDU-Other Teachers And Instructors | 2,345 (0.8) | 588 | 12,970 (0.8) | 1,496 | 0.02 |
| EDU-Archivists, Curators, And Museum Technicians |  |  | 1,299 (0.08) | 372 | -0.08 |
| EDU-Librarians And Media Collections Specialists |  |  | 2,251 (0.1) | 495 | -0.14 |
| EDU-Library Technicians |  |  | 410 (0.02) | 241 | -0.02 |
| EDU-Teaching Assistants | 8,540 (2.9) | 1,318 | 32,420 (2.0) | 2,662 | 0.97 |
| EDU-Other Educational Instruction and Library Workers | 324 (0.1) | 196 | 2,988 (0.2) | 588 | -0.07 |
| **Medical workers** | 9,814 (3.4) | 1,272 | 325,651 (19.6) | 7,577 |  |
| MED-Chiropractors | 155 (0.05) | 169 | 1,445 (0.09) | 570 | -0.03 |
| MED-Dentists |  |  | 3,969 (0.2) | 815 | -0.24 |
| MED-Dietitians And Nutritionists | 125 (0.04) | 130 | 3,521 (0.2) | 674 | -0.17 |
| MED-Optometrists |  |  | 1,602 (0.1) | 468 | -0.1 |
| MED-Pharmacists |  |  | 10,349 (0.6) | 1,264 | -0.62 |
| MED-Physician Assistants |  |  | 8,155 (0.5) | 1,036 | -0.49 |
| MED-Podiatrists |  |  | 112 (0.007) | 111 | -0.01 |
| MED-Occupational Therapists | 163 (0.06) | 160 | 6,365 (0.4) | 887 | -0.33 |
| MED-Physical Therapists | 178 (0.06) | 143 | 10,287 (0.6) | 1,162 | -0.56 |
| MED-Radiation Therapists |  |  | 489 (0.03) | 199 | -0.03 |
| MED-Recreational Therapists | 141 (0.05) | 107 | 567 (0.03) | 334 | 0.01 |
| MED-Respiratory Therapists | 33 (0.01) | 34 | 3,899 (0.2) | 773 | -0.22 |
| MED-Speech-Language Pathologists |  |  | 9,851 (0.6) | 1,138 | -0.59 |
| MED-Other Therapists | 260 (0.09) | 185 | 6,239 (0.4) | 914 | -0.29 |
| MED-Veterinarians |  |  | 3,720 (0.2) | 758 | -0.22 |
| MED-Registered Nurses | 1,686 (0.6) | 467 | 128,928 (7.8) | 3,915 | -7.19 |
| MED-Nurse Anesthetists |  |  | 1,483 (0.09) | 475 | -0.09 |
| MED-Audiologists |  |  | 1,058 (0.06) | 435 | -0.06 |
| MED-Nurse Practitioners, And Nurse Midwives |  |  | 13,171 (0.8) | 1,305 | -0.79 |
| MED-Physicians |  |  | 18,156 (1.1) | 1,315 | -1.09 |
| MED-Surgeons |  |  | 513 (0.03) | 219 | -0.03 |
| MED-Acupuncturists |  |  | 268 (0.02) | 166 | -0.02 |
| MED-Dental Hygienists | 233 (0.08) | 220 | 8,591 (0.5) | 1,083 | -0.44 |
| MED-Healthcare Diagnosing Or Treating Practitioners, All Other |  |  | 85 (0.005) | 85 | -0.01 |
| MED-Clinical Laboratory Technologists And Technicians | 230 (0.08) | 156 | 6,278 (0.4) | 932 | -0.3 |
| MED-Cardiovascular Technologists And Technicians | 148 (0.05) | 109 | 1,549 (0.1) | 644 | -0.04 |
| MED-Diagnostic Medical Sonographers | 56 (0.02) | 62 | 3,161 (0.2) | 567 | -0.17 |
| MED-Radiologic Technologists And Technicians | 297 (0.1) | 204 | 6,798 (0.4) | 860 | -0.31 |
| MED-Magnetic Resonance Imaging Technologists |  |  | 606 (0.04) | 233 | -0.04 |
| MED-Nuclear Medicine Technologists And Medical Dosimetrists | 75 (0.03) | 80 | 284 (0.02) | 117 | 0.01 |
| MED-Emergency Medical Technicians | 96 (0.03) | 70 | 2,440 (0.1) | 938 | -0.11 |
| MED-Paramedics |  |  | 2,168 (0.1) | 677 | -0.13 |
| MED-Pharmacy Technicians | 1,272 (0.4) | 501 | 15,194 (0.9) | 1,798 | -0.48 |
| MED-Psychiatric Technicians | 143 (0.05) | 98 | 1,587 (0.1) | 465 | -0.05 |
| MED-Surgical Technologists |  |  | 2,926 (0.2) | 616 | -0.18 |
| MED-Veterinary Technologists And Technicians | 826 (0.3) | 353.9635 | 6,375 (0.4) | 1,093 | -0.1 |
| MED-Dietetic Technicians And Ophthalmic Medical Technicians | 326 (0.1) | 179.9661 | 1,222 (0.1) | 394 | 0.04 |
| MED-Licensed Practical And Licensed Vocational Nurses | 2,740 (0.9) | 649 | 19,632 (1.2) | 1,911 | -0.25 |
| MED-Medical Records Specialists | 326 (0.1) | 204 | 5,876 (0.4) | 1,037 | -0.24 |
| MED-Opticians, Dispensing | 266 (0.09) | 160 | 2,588 (0.2) | 890 | -0.07 |
| MED-Miscellaneous Health Technologists And Technicians |  |  | 2,080 (0.1) | 492 | -0.13 |
| MED-Other Healthcare Practitioners And Technical Occupations | 39 (0.01) | 41 | 2,064 (0.1) | 460 | -0.11 |

Abbreviation: N, number; SE, standard errors; SOC, standard occupational classification

Note: a) Occupation information was collected using 2018 SOC codes; b) Most frequent major occupations of each poverty and non-poverty group are sales and related workers, food preparation and serving related workers, office and administrative support workers, healthcare support workers, cleaners and grounds maintenance workers, managers, educational workers, and medical workers; c) Poverty (<100%) vs non-poverty (≥100%) was defined using income-to-poverty ratio; d) We highlighted specific jobs in red where a difference of weighted percentages between poverty and non-poverty groups was >1.5% and also highlighted ones in blue where the difference was <-1.5%, indicating that the item was more frequent in women without poverty; e) Weighted estimates; f) Weighted percentage of poverty group - Weighted percentage of non-poverty group
