# Supplementary material for: Major Occupations and Private Insurance of Working Postpartum Women in Poverty in the United States, 2019
Source: Womens Health Rep (New Rochelle). 2023 Nov 14;4(1):497–505. doi: 10.1089/whr.2023.0042 (PMC10615075; doi:10.1089/whr.2023.0042)
Supplement: Supplemental data [file Suppl_TableS2-S5.docx]

**Table S2. Distributions of age and race according to poverty status ^a^ in each specific job ^b^ among US working postpartum women**

|  | **Age ^c^** | | | **Race ^d^** | | | | | | | | |
| --- | --- | --- | --- | --- | --- | --- | --- | --- | --- | --- | --- | --- |
|  | **Total** | **Poverty** | **Non-poverty** | **Total** | | | **Poverty** | | | **Non-poverty** | | |
| **Characteristics** |  |  |  | **White** | **Black or African American** | **Others** | **White** | **Black or African American** | **Others** | **White** | **Black or African American** | **Others** |
| SAL-Cashiers | 25.6 (0.3) | 25.9 (0.4) | 25.5 (0.3) | 54,172 (52.9) | 29,973 (29.3) | 18,310 (17.9) | 19,511 (49.0) | 14,935 (37.5) | 5,351 (13.4) | 34,661 (55.3) | 15,038 (24.0) | 12,959 (20.7) |
| EAT-Cooks | 29.0 (0.6) | 27.9 (0.9) | 29.8 (0.8) | 19,268 (58.4) | 6,460 (19.6) | 7,273 (22.0) | 8,772 (65.9) | 2,785 (20.9) | 1,749 (13.1) | 10,496 (53.3) | 3,675 (18.7) | 5,524 (28.0) |
| EAT-Food Preparation Workers | 27.7 (0.8) | 27.4 (1.6) | 28.0 (0.8) | 15,046 (69.2) | 2,590 (11.9) | 4,108 (18.9) | 5,136 (65.0) | 1,009 (12.8) | 1,756 (22.2) | 9,910 (71.6) | 1,581 (11.4) | 2,352 (17.0) |
| EAT-Fast Food And Counter Workers | 24.9 (0.6) | 25.4 (1.0) | 27.8 (0.9) | 8,556 (57.5) | 3,308 (22.0) | 3,080 (20.5) | 3,817 (61.3) | 1,590 (25.6) | 816 (13.1) | 4,839 (54.9) | 1,718 (19.5) | 2,264 (25.7) |
| EAT-Waiters And Waitresses | 26.8 (0.4) | 24.8 (0.4) | 27.8 (0.5) | 48,073 (73.5) | 8,099 (12.4) | 9,273 (14.2) | 15,866 (72.5) | 3,418 (15.6) | 2,588 (11.8) | 32,207 (73.9) | 4,681 (10.7) | 6,685 (15.3) |
| HLS-Personal Care Aides | 29.4 (0.4) | 28.3 (0.8) | 30.1 (0.5) | 20,141 (48.4) | 13,908 (33.4) | 7,570 (18.2) | 6,236 (42.4) | 5,783 (39.3) | 2,687 (18.3) | 13,905 (51.7) | 8,125 (30.2) | 4,883 (18.1) |
| HLS-Nursing Assistants | 28.5 (0.3) | 27.9 (0.6) | 28.7 (0.4) | 30,875 (49.5) | 22,151 (35.5) | 9,334 (15.0) | 5,525 (31.8) | 9,303 (53.6) | 2,520 (14.5) | 25,350 (56.3) | 12,848 (28.5) | 6,814 (15.1) |
| CLN-Maids And Housekeeping Cleaners | 31.8 (0.5) | 30.9 (0.8) | 32.6 (0.7) | 22,957 (56.0) | 6,963 (17.0) | 11,110 (27.1) | 9,843 (54.0) | 4,299 (23.6) | 4,075 (22.4) | 13,114 (57.5) | 2,664 (11.7) | 7,035 (30.8) |
| CLN-Janitors And Building Cleaners | 30.6 (0.7) | 27.9 (0.7) | 31.9 (0.9) | 12,748 (56.7) | 5,184 (23.0) | 4,559 (20.3) | 3,290 (45.9) | 2,603 (36.3) | 1,273 (17.8) | 9,458 (61.7) | 2,581 (16.8) | 3,286 (21.4) |
| MGR-Financial Managers | 32.4 (0.4) | 28.5 (4.4) | 32.4 (0.4) | 21,480 (78.9) | 2,531 (9.3) | 3,217 (11.8) | 124 (100.0) |  |  | 21,356 (78.8) | 2,531 (9.3) | 3,217 (11.9) |
| MGR-Other Managers | 32.9 (0.4) | 27.6 (1.0) | 33.3 (0.4) | 35,595 (70.6) | 7,183 (14.3) | 7,611 (15.1) | 1,535 (47.7) | 1,127 (35.0) | 555 (17.3) | 34,060 (72.2) | 6,056 (12.8) | 7,056 (15.0) |
| EDU-Elementary And Middle School Teachers | 32.6 (0.2) | 31.6 (1.7) | 32.7 (0.2) | 83,582 (83.3) | 9,504 (9.5) | 7,228 (7.2) | 1,772 (51.2) | 1,425 (41.2) | 263 (7.6) | 81,810 (84.5) | 8,079 (8.3) | 6,965 (7.2) |
| EDU-Secondary School Teachers | 33.0 (0.3) | 28.8 (1.0) | 33.2 (0.3) | 29,158 (84.5) | 1,830 (5.3) | 3,526 (10.2) | 956 (76.8) |  | 289 (23.2) | 28,202 (84.8) | 1,830 (5.5) | 3,237 (9.7) |
| MED-Registered Nurses | 32.0 (0.2) | 32.8 (1.5) | 32.0 (0.2) | 100,882 (77.2) | 13,411 (10.3) | 16,321 (12.5) | 1,247 (74.0) | 217 (12.9) | 222 (13.2) | 99,635 (77.3) | 13,194 (10.2) | 16,099 (12.5) |

Abbreviation: N, number; SE, standard errors; SOC, standard occupational classification

Note: a) Poverty (<100%) vs non-poverty (≥100%) was defined using income-to-poverty ratio; b) Occupation information was collected using 2018 SOC codes; c) Weighted mean (Standard error of mean); d) Weighted frequency (Weighted percentage)

**Table S3. Total person’s incomes and income-to-poverty ratios by poverty status ^a^ in each specific job ^b^ among US working postpartum women**

|  | **Total person's income ^c^** | | | **Income-to-poverty ratio ^c^** | | |
| --- | --- | --- | --- | --- | --- | --- |
| **Characteristics** | **Total** | **Poverty** | **Non-poverty** | **Total** | **Poverty** | **Non-poverty** |
| SAL-Cashiers | 13,363.0 (810.1) | 7,672.3 (390.2) | 16,978.0 (1,228.3) | 167.5 (4.9) | 43.0 (2.0) | 240.7 (6.1) |
| EAT-Cooks | 15,284.0 (901.6) | 8,039.6 (661.4) | 20,178.0 (1,170.8) | 165.0 (10.2) | 51.3 (3.0) | 237.5 (11.8) |
| EAT-Food Preparation Workers | 12,308.0 (1,068.6) | 7,529.4 (1,049.4) | 15,035.0 (1,342.7) | 168.0 (12.6) | 53.0 (5.3) | 233.5 (14.4) |
| EAT-Fast Food And Counter Workers | 11,292.0 (1,390.1) | 7,165.1 (1,029.4) | 14,204.0 (2,201.0) | 170.4 (18.1) | 43.1 (4.2) | 258.7 (19.8) |
| EAT-Waiters And Waitresses | 15,211.0 (564.0) | 6,244.0 (462.5) | 18,764.0 (798.2) | 187.1 (6.7) | 53.7 (2.8) | 252.3 (7.5) |
| HLS-Personal Care Aides | 18,934.0 (884.5) | 11,619 (894.1) | 22,930.0 (1,290.5) | 168.7 (9.4) | 57.9 (3.5) | 229.2 (10.3) |
| HLS-Nursing Assistants | 20,137.0 (751.8) | 11,685 (899.0) | 23,394.0 (898.7) | 201.7 (8.5) | 60.2 (3.1) | 254.8 (9.0) |
| CLN-Maids And Housekeeping Cleaners | 14,745.0 (1,585.5) | 7,989.6 (553.2) | 20,140.0 (2,735.0) | 144.0 (7.7) | 44.2 (2.3) | 222.9 (9.8) |
| CLN-Janitors And Building Cleaners | 14,526.0 (1,105.6) | 7,716.9 (1,143.6) | 17,710.0 (1,424.1) | 165.3 (9.0) | 40.9 (5.4) | 222.0 (7.3) |
| MGR-Financial Managers | 79,039.0 (4,629.3) | 23,819.0 (1,885.4) | 79,291.0 (4,635.5) | 404.6 (10.0) | 74.0 (0.0) | 405.7 (9.8) |
| MGR-Other Managers | 75,065.0 (3,666.5) | 13,229.0 (1,293.1) | 79,282 (3,884.5) | 393.6 (8.7) | 55.3 (3.7) | 416.6 (7.8) |
| EDU-Elementary And Middle School Teachers | 44,553.0 (994.0) | 9,722.4 (1,307.5) | 45,797.0 (955.0) | 389.5 (6.0) | 54.0 (4.1) | 401.5 (5.4) |
| EDU-Secondary School Teachers | 48,872.0 (1,479.7) | 13,884.0 (3,328.1) | 50,182.0 (1,428.7) | 396.2 (10.1) | 56.9 (12.2) | 408.9 (7.9) |
| MED-Registered Nurses | 56,033.0 (1,295.9) | 10,573.0 (1,864.6) | 56,627.0 (1,306.8) | 402.8 (3.9) | 59.1 (4.2) | 407.3 (3.6) |

Abbreviation: N, number; SE, standard errors; SOC, standard occupational classification

Note: a) Poverty (<100%) vs non-poverty (≥100%) was defined using income-to-poverty ratio; b) Occupation information was collected using 2018 SOC codes; c) Weighted mean (Standard errors of mean)

**Table S4. Weekly working hours and working weeks during past 12 months of specific jobs ^a^ according to poverty status ^b^, among US working postpartum women**

|  | **Usual hours worked per week during past 12 months ^c^** | | | **Weeks worked during past 12 months ^c^** | | |
| --- | --- | --- | --- | --- | --- | --- |
| **Characteristics** | **Total** | **Poverty** | **Non-poverty** | **Total** | **Poverty** | **Non-poverty** |
| SAL-Cashiers | 29.1 (0.4) | 27.1 (0.6) | 30.3 (0.6) | 36.6 (0.9) | 31.7 (1.5) | 39.7 (0.9) |
| EAT-Cooks | 32.8 (0.9) | 30.0 (1.4) | 34.8 (1.1) | 38.1 (1.3) | 32.6 (2.0) | 41.9 (1.5) |
| EAT-Food Preparation Workers | 28.7 (1.2) | 26.9 (2.2) | 29.7 (1.2) | 37.0 (1.3) | 34.2 (2.2) | 38.6 (1.7) |
| EAT-Fast Food And Counter Workers | 28.2 (1.2) | 27.2 (1.5) | 28.8 (1.8) | 31.3 (2.1) | 22.6 (3.4) | 37.4 (2.8) |
| EAT-Waiters And Waitresses | 28.9 (0.5) | 26.2 (0.6) | 30.2 (0.6) | 38.2 (0.8) | 33.2 (1.7) | 40.7 (1.0) |
| HLS-Personal Care Aides | 34.2 (0.8) | 32.1 (1.4) | 35.3 (1.0) | 40.1 (1.1) | 33.1 (2.2) | 43.9 (1.1) |
| HLS-Nursing Assistants | 34.6 (0.6) | 31.9 (1.3) | 35.6 (0.7) | 41.7 (0.9) | 35.2 (1.8) | 44.2 (0.9) |
| CLN-Maids And Housekeeping Cleaners | 30.4 (0.8) | 28.8 (1.3) | 31.6 (1.0) | 39.0 (1.2) | 35.2 (1.7) | 42.0 (1.5) |
| CLN-Janitors And Building Cleaners | 30.1 (1.1) | 26.0 (2.5) | 32.1 (1.4) | 36.2 (1.6) | 27.2 (3.1) | 40.4 (2.0) |
| MGR-Financial Managers | 41.3 (0.6) | 31.3 (11.1) | 41.3 (0.6) | 48.6 (0.6) | 39.2 (16.3) | 48.7 (0.6) |
| MGR-Other Managers | 39.9 (0.5) | 33.3 (2.6) | 40.4 (0.4) | 46.7 (0.6) | 41.2 (3.8) | 47.0 (0.6) |
| EDU-Elementary And Middle School Teachers | 38.9 (0.4) | 29.1 (3.1) | 39.2 (0.4) | 44.9 (0.5) | 41.9 (3.8) | 45.0 (0.5) |
| EDU-Secondary School Teachers | 39.9 (0.7) | 36.0 (11.9) | 40.0 (0.6) | 44.2 (0.9) | 31.8 (7.3) | 44.7 (0.9) |
| MED-Registered Nurses | 34.7 (0.4) | 26.3 (2.7) | 34.8 (0.4) | 46.9 (0.4) | 31.5 (6.2) | 47.1 (0.3) |

Abbreviation: N, number; SE, standard errors; SOC, standard occupational classification

Note: a) Occupation information was collected using 2018 SOC codes; b) Poverty (<100%) vs non-poverty (≥100%) was defined using income-to-poverty ratio; c) Weighted mean (Standard errors of mean)

**Table S5. Receipt of private insurance according to poverty status ^a^ in each specific job ^b^ among US working postpartum women**

|  | **Insurance through an employer/union ^c^** | | | | | | **Private health insurance ^c^** | | | | | |
| --- | --- | --- | --- | --- | --- | --- | --- | --- | --- | --- | --- | --- |
|  | **Total** | | **Poverty** | | **Non-poverty** | | **Total** | | **Poverty** | | **Non-poverty** | |
| **Characteristics** | **Yes** | **No** | **Yes** | **No** | **Yes** | **No** | **Yes** | **No** | **Yes** | **No** | **Yes** | **No** |
| SAL-Cashiers | 26,634 (26.0) | 75,821 (74.0) | 5,339 (13.4) | 34,458 (86.6) | 21,295 (34.0) | 41,363 (66.0) | 30,999 (30.3) | 71,456 (69.7) | 6,119 (15.4) | 33,678 (84.6) | 24,880 (39.7) | 37,778 (60.3) |
| EAT-Cooks | 9,513 (28.8) | 23,488 (71.2) | 1,546 (11.6) | 11,760 (88.4) | 7,967 (40.5) | 11,728 (59.5) | 11,237 (34.1) | 21,764 (65.9) | 2,161 (16.2) | 11,145 (83.8) | 9,076 (46.1) | 10,619 (53.9) |
| EAT-Food Preparation Workers | 6,422 (29.5) | 15,322 (70.5) | 837 (10.6) | 7,064 (89.4) | 5,585 (40.3) | 8,258 (59.7) | 8,512 (39.1) | 13,232 (60.9) | 1,588 (20.1) | 6,313 (79.9) | 6,924 (50.0) | 6,919 (50.0) |
| EAT-Fast Food And Counter Workers | 3,820 (25.4) | 11,224 (74.6) | 738 (11.9) | 5,485 (88.1) | 4,354 (34.1) | 8,430 (65.9) | 4,480 (29.8) | 10,564 (70.2) | 807 (13.0) | 5,416 (87.0) | 3,673 (41.6) | 5,148 (58.4) |
| EAT-Waiters And Waitresses | 17,631 (26.9) | 47,814 (73.1) | 5,358 (17.0) | 26,118 (83.0) | 13,830 (31.7) | 29,743 (68.3) | 23,485 (35.9) | 41,960 (64.1) | 4,213 (19.3) | 17,659 (80.7) | 19,272 (44.2) | 24,301 (55.8) |
| HLS-Personal Care Aides | 12,797 (30.7) | 28,822 (69.3) | 1,666 (11.3) | 13,040 (88.7) | 11,131 (41.4) | 15,782 (58.6) | 15,220 (36.6) | 26,399 (63.4) | 2,269 (15.4) | 12,437 (84.6) | 12,951 (48.1) | 13,962 (51.9) |
| HLS-Nursing Assistants | 28,523 (45.7) | 33,837 (54.3) | 3,611 (20.8) | 13,737 (79.2) | 24,912 (55.3) | 20,100 (44.7) | 32,183 (51.6) | 30,177 (48.4) | 3,634 (20.9) | 13,714 (79.1) | 28,549 (63.4) | 16,463 (36.6) |
| CLN-Maids And Housekeeping Cleaners | 7,663 (18.7) | 33,367 (81.3) | 1,123 (6.2) | 17,094 (93.8) | 6,540 (28.7) | 16,273 (71.3) | 10,011 (24.4) | 31,019 (75.6) | 1,339 (7.4) | 16,878 (92.6) | 8,672 (38.0) | 14,141 (62.0) |
| CLN-Janitors And Building Cleaners | 6,493 (28.9) | 15,998 (71.1) | 494 (6.9) | 6,672 (93.1) | 5,999 (39.1) | 9,326 (60.9) | 7,344 (32.7) | 15,147 (67.3) | 1,086 (15.2) | 6,080 (84.8) | 6,258 (40.8) | 9,067 (59.2) |
| MGR-Financial Managers | 23,442 (86.1) | 3,786 (13.9) | 124 (100.0) |  | 23,318 (86.0) | 3,786 (14.0) | 24,733 (90.8) | 2,495 (9.2) | 124 (100.0) |  | 24,609 (90.8) | 2,495 (9.2) |
| MGR-Other Managers | 40,102 (79.6) | 10,287 (20.4) | 58 (1.8) | 3,159 (98.2) | 40,044 (84.9) | 7,128 (15.1) | 43,476 (86.3) | 6,913 (13.7) | 70 (2.2) | 3,147 (97.8) | 43,406 (92.0) | 3,766 (8.0) |
| EDU-Elementary And Middle School Teachers | 90,214 (89.9) | 10,100 (10.1) | 1,555 (44.9) | 1,905 (55.1) | 88,659 (91.5) | 8,195 (8.5) | 94,424 (94.1) | 5,890 (5.9) | 1,555 (44.9) | 1,905 (55.1) | 92,869 (95.9) | 3,985 (4.1) |
| EDU-Secondary School Teachers | 30,108 (87.2) | 4,406 (12.8) | 117 (9.4) | 1,128 (90.6) | 29,991 (90.1) | 3,278 (9.8) | 30,858 (89.4) | 3,656 (10.6) | 117 (9.4) | 1,128 (90.6) | 30,741 (92.4) | 2,528 (7.6) |
| MED-Registered Nurses | 114,596 (87.7) | 16,018 (12.3) | 763 (45.3) | 923 (54.7) | 113,833 (88.3) | 15,095 (11.7) | 121,936 (93.4) | 8,678 (6.6) | 1,075 (63.8) | 611 (36.2) | 120,861 (93.7) | 8,067 (6.3) |

Abbreviation: N, number; SE, standard errors; SOC, standard occupational classification

Note: a) Poverty (<100%) vs non-poverty (≥100%) was defined using income-to-poverty ratio; b) Occupation information was collected using 2018 SOC codes; c) Weighted frequency (Weighted percentage) geelskpercenta
